# Case of Convulsions from Organic Disease of the Brain

**Published:** 1868-11

**Authors:** S. T. Odell

**Affiliations:** St. Louis, Kansas


ARTICLE XLVII.

### CASE OF CONVULSIONS FROM ORGANIC DISEASE OF THE BRAIN.

By S. T. ODELL, M.D., St. Louis, Kansas.

Although the following case, which occurred in my practice at Atwood, Ind., loses much of the interest which it would otherwise possess from the fact that it passed from under observation prior to its termination, yet it is reported, with the belief that it will be of some slight value as a contribution to the literature of organic disease of the brain.

December 29th, 1867, I was called to see Jerry L., aged 35, farm laborer, unmarried. Weight about 160 pounds; muscular system well developed. I obtained from friends present the following history of his case up to the time I was called:—

He was struck in right eye by a snow-ball in the winter of 1860, and was confined to his bed two days in consequence. Those now with him were not present at that time, and are unable to state the character of his sickness then, and he cannot himself remember anything more about it than that he was struck, and afterwards *awaked* with a very much swollen eye, and that after the swelling subsided he had lost the use of his right eye.

From the date of his getting up from this injury he was able, as before, to attend to all his duties as general farm laborer, and was in the enjoyment of robust health up to December 1st, 1867. On this day, while engaged in the light work of feeding cattle in the farm-yard, he suddenly became conscious of the fact that his sight was failing him, and although he made all possible haste to the house, so rapidly did loss of sight supervene, that it was with difficulty that he discovered his way. On reaching the house he laid down, and in less than two hours from the time of his leaving the farm-yard his left, and hitherto useful, eye was as useless for sight as had been the other for years.

Simultaneously with this loss of vision came on a severe shaking fit, resembling somewhat the rigors of intermittent fever. He felt, however, no chilliness during the course of the convulsive movements. After several minutes this shaking subsided, leaving him with a heavy headache for about two hours, when, to use his own expression, he "felt all right—good as ever—only couldn't see." At intervals ranging from one to twenty-four hours he had returns of these convulsive movements, each recurring paroxysm being more severe and protracted. At times they affected most the muscles of one side of the body, at other times the opposite side, and again they would severely shake the entire body. The statement that these convulsions at times affected but one side of the body is made on the strength of the positive and repeated assertions of his friends, for at no time during my personal observation of the case did it occur.

At 3 o'clock P.M., December 29th, he was attacked with convulsions more violently than usual, and, as they continued with but momentary respite until he seemed nearly worn out, at dark I was sent for, and arrived about 8 o'clock P.M. When I arrived the patient was lying on a bed and shaking so violently as to agitate the bedstead, and even occasionally to jar the entire house.

The attacks varied from ten seconds to two minutes in duration, with an interval between them of from thirty to sixty seconds. Patient perfectly conscious both during the convulsions and intervals. Pulse, as counted in the interval, 70 per minute. Tongue clean, of normal appearance, and protruded without hesitation or difficulty. Taste normal. Hearing perfect in the left ear, but in the right much impaired; on inquiry, it was ascertained that this fact had not been noticed prior to this evening. I regret that want of appliances, as well as a want of skill in their use, to which I must confess, prevented my making an ophthalmoscopic examination of the eyes at this, or any other time during the progress of the case. The right eye, which was injured by the snow-ball seven years ago, was normal in external appearance; pupil, however, contracted to the size of an ordinary pin-head. The pupil of the left eye was widely dilated, and would not respond to the light. Patient stated that he could distinguish daylight from darkness with this eye; this may be doubted, as he was unable to tell any difference in the light of the room when the lamp was held immediately before his eyes and when it was withdrawn from the room.

Appetite but little impaired during the entire time that he was under observation; his bowels were constipated. He stated that his "spells," as he termed them, were invariably preceded, accompanied, and followed by a heavy, dull aching in the back part of his head, his neck and back; and that the "shaking" seemed to commence in the back part of his head, and extend thence all over him; when, in fact, the invasion of a paroxysm was marked to the bystander by a twitching of the muscles of the extremities, which was speedily followed by the motions immediately described.

At the commencement of each attack the forearms were semiflexed upon the arms, the hands semiflexed upon the forearms, thighs slightly bent upon abdomen, and the legs upon the thighs. These relative positions of the members were not changed by the convulsions. The trunk was only slightly bent forward, and at no time was there either opisthotonos or lateral contortion of the body. During a convulsion the flexors and extensors seemed so evenly balanced in the amount of motor influence alternately received, that neither the hands or feet would move more than six inches under the influence of one set of muscles, until they were violently jerked in the other direction by the opposing set; while the motion of the lower portion of the trunk backward and forward was, although limited in extent of space, almost inconceivably rapid. The muscles of the face were affected but slightly, if at all, and respiration was not interfered with to any notable degree.

I am thus particular in describing these convulsions because they were unlike anything I had heretofore seen.

In intellect the patient was always somewhat obtuse, without, however, being an imbecile. On inquiry, I learned that since his attack on December 1st, the patient had been for a short time under the treatment of Dr. Gray, of Palestine, Ind., who had assaulted the malady with the whole battery of antispasmodics, and without making an impression, and afterwards of a quack "Indian Doctor" of Warsaw, Ind., the nature of whose medication in the case I was unable to learn; its result, however, was, to say the least, not beneficial.

As I deemed it difficult, if not impossible, to arrive unaided at a satisfactory diagnosis upon which to base treatment, I suggested the propriety of sending for counsel; to this the friends of the patient would not accede, alleging that they had already had in employ one regular physician, and "The Indian Doctor," to no avail, and that they were not disposed to go to any further expense in the treatment of a case apparently so hopeless. At this point my interest in observing the course of the disease alone prevented my immediate withdrawal from any further connection with the case.

As the idea of chronic inflammation along the base of the brain seemed the most plausible in accounting for the symptoms present, I applied a blister at the nape of the neck, and gave a sharp purgative, promising to return next morning and continue treatment. The patient complained of pain in the limbs and back, and as I was perhaps unduly cautious as to the use of opium in his case (as I was uncertain as to the correctness of my diagnosis), I left a few doses of ext. hyoscyamus, to be taken at intervals through the night.

*January 30th.*—Received word that Jerry was better, and that I need not visit him. Discharged because I had not held out hopes of a cure.

*December 13th, 1868.*—As I was passing on my way to another patient, I stopped to see Jerry. He has received no treatment since my visit. Convulsions still recurring every few hours, or more often, and he is manifestly losing flesh and strength. Appetite good. Pulse *40 per minute.* Both pupils moderately dilated.

*March 17th.*—Jerry no better, his longest respite from convulsions being twelve to eighteen hours. Pulse 45 per minute. In addition to convulsions, now complains of occasional dyspnœa; says he "can hardly get his breath sometimes for half an hour at a time." Has had no treatment of any kind since December 29th.

*July 8th.*—Saw Jerry at work washing clothes, still blind, still suffering from occasional difficulty of breathing, and still shaking every few hours. Pulse 60 per minute, and general health much improved.

Shortly after this date I removed to my present location, and lost sight of the case. Since its occurrence I have read with especial interest all the reported cases of tumor of the brain upon which I could lay my hands, yet I find in none of them an analogous case. If chronic inflammation along the base of the brain caused the symptoms observed, the next query would relate to the cause of the inflammation: The blow from the snow-ball seven years ago? I know of nothing else in the history of the case that could stand in a causative relation to such a supposed condition.

Of the cranial nerves, only the 2d and auditory division of the 7th (on the right side) were, as per evidence of the symptoms, affected; unless, indeed, the occasional attacks of dyspnœa complained of be referable to perverted action of the pneumogastric.

But for the evidence of organic disease furnished by these nerves, I should have felt no hesitancy in pronouncing the affection a case of *Convulsive Tumor*, as described by Dr. W. A. Hammond (*New York Med. Jour.*, June, 1867—Ranking's *Hf.-Yearly Abs.*, Vol. XVI., p. 48, *et seq.*).

---

Abortion.—Dr. Fordyce Barker places great faith in chlorate of potash. This was first suggested by Sir James Y. Simpson, on the ground that its oxygen producing power would be beneficial in fatty placenta. Whatever might be the truth of this chemical theory, clinical experience has convinced Mr. Barker of the value of this remedy. Patients themselves notice its effect upon the movements of the fœtus. He relates several remarkable cases of success with this remedy after repeated abortions.—*Dominion Medical Journal.*

